# Pfs230 Domain 7 is targeted by a potent malaria transmission-blocking monoclonal antibody

**DOI:** 10.1038/s41541-023-00784-x

**Published:** 2023-12-12

**Authors:** Maartje R. Inklaar, Roos M. de Jong, Ezra T. Bekkering, Hikaru Nagaoka, Felix L. Fennemann, Karina Teelen, Marga van de Vegte-Bolmer, Geert-Jan van Gemert, Rianne Stoter, C. Richter King, Nicholas I. Proellochs, Teun Bousema, Eizo Takashima, Takafumi Tsuboi, Matthijs M. Jore

**Affiliations:** 1 Department of Medical Microbiology, Radboud University Medical Center, Nijmegen, The Netherlands; 2 Division of Malaria Research, Proteo-Science Center, Ehime University, Matsuyama, Japan; 3 Department of Tumor Immunology, Radboud University Medical Center, Nijmegen, The Netherlands; 4 Center for Vaccine Innovation and Access, PATH, Washington, DC USA; 5 Division of Cell-Free Sciences, Proteo-Science Center, Ehime University, Matsuyama, Japan

**Keywords:** Protein vaccines, Malaria

## Abstract

Malaria transmission-blocking vaccines (TBVs) aim to induce antibodies that block *Plasmodium* parasite development in the mosquito midgut, thus preventing mosquitoes from becoming infectious. While the Pro-domain and first of fourteen 6-Cysteine domains (Pro-D1) of the *Plasmodium* gamete surface protein Pfs230 are known targets of transmission-blocking antibodies, no studies to date have discovered other Pfs230 domains that are functional targets. Here, we show that a murine monoclonal antibody (mAb), 18F25.1, targets Pfs230 Domain 7. We generated a subclass-switched complement-fixing variant, mAb 18F25.2a, using a CRISPR/Cas9-based hybridoma engineering method. This subclass-switched mAb 18F25.2a induced lysis of female gametes in vitro. Importantly, mAb 18F25.2a potently reduced *P. falciparum* infection of *Anopheles stephensi* mosquitoes in a complement-dependent manner, as assessed by standard membrane feeding assays. Together, our data identify Pfs230 Domain 7 as target for transmission-blocking antibodies and provide a strong incentive to study domains outside Pfs230Pro-D1 as TBV candidates.

## Introduction

After a decade of considerable gains, the decline in malaria cases and deaths has completely stalled in recent years^1^, underscoring the need for novel interventions including highly effective vaccines. *Plasmodium* parasites are the causative agents of malaria, with *Plasmodium falciparum* being responsible for the vast majority of deaths. Transmission of *Plasmodium* parasites through the human population occurs via *Anopheles* mosquitoes and commences with the uptake of gametocyte-infected erythrocytes from an infected human during a mosquito blood meal. In the mosquito midgut, female and male gametocytes activate and develop into gametes that egress from the erythrocyte. Male gametes fertilize female gametes, resulting in zygotes that differentiate into ookinetes capable of traversing the mosquito midgut epithelial layer. After traversal, oocysts are formed, in which sporozoites develop that migrate to the salivary glands and render a mosquito infectious to humans upon a next bite.

Malaria transmission-blocking vaccines (TBVs) are designed to induce antibodies against antigens on the parasite or mosquito midgut surface. When taken up together with gametocytes, these antibodies can interrupt sexual development in the mosquito midgut and reduce parasite transmission. As a consequence of this vaccine-induced transmission reducing activity (TRA), TBVs can limit the number of infectious mosquitoes and play an important role in efforts to eliminate malaria^2^.

**Fig. 1:**
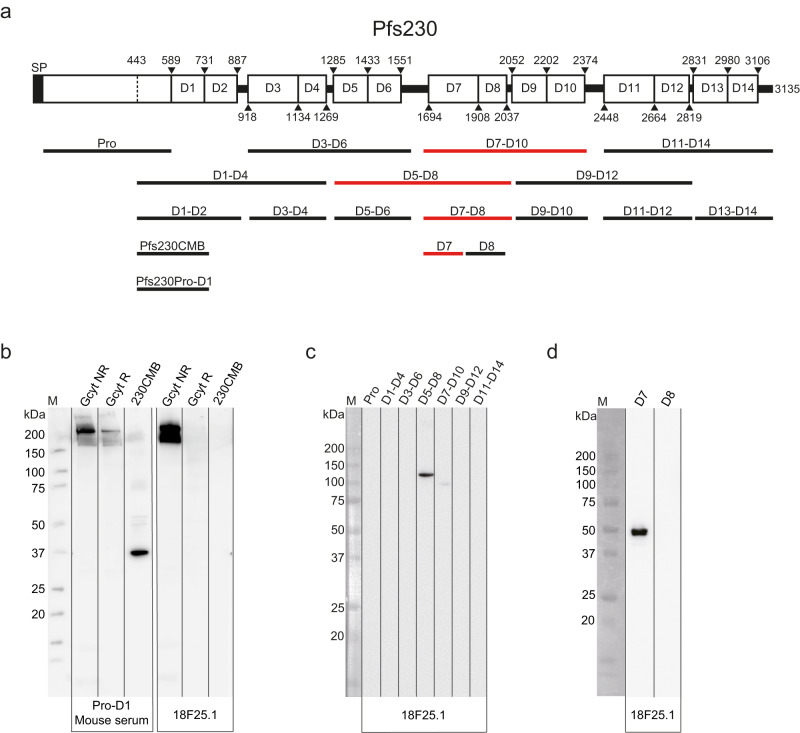
mAb 18F25 targets Pfs230D7. **a** Schematic illustration of the Pfs230 domains and design of the constructs. Domain, linker segments and fragment boundaries are indicated with amino acid numbers (Supplementary Table S1)^3^. Pro is an abbreviation for the Pro-domain, which contains cleavage sites (mapped between 477–487 and 523–555^44^) that are cleaved during gamete formation. SP is an abbreviation for signal peptide. The constructs in red are recognized by mAb 18F25.1 and all contain D7. **b** Western blot with *P. falciparum* NF54 gametocyte extract (Gcyt) under reduced (R) and non-reduced (NR) conditions and 20 ng of recombinant Pfs230CMB (aa 444–730) under NR conditions^34^. All samples were separated on one gel and transferred to a blot. The blot was cut in two parts that were incubated with mAb 18F25 IgG1 (18F25.1) and serum from mice immunized with Pro-D1 (aa 443–736)^35^, respectively. Serum raised against Pfs230Pro-D1 was included as positive control and recognized both 230CMB and native Pfs230. Pro-D1 and 230CMB are abbreviations for Pfs230Pro-D1 and Pfs230CMB, respectively. **c**, **d** Western blots with recombinant Pfs230 fragments shown in (**a**) that were incubated with mAb 18F25.1. Note that the recombinant fragments were expressed with a glutathione S-transferase (GST) tag and therefore migrate as larger products.

Pfs230D1-EPA is the most clinically advanced TBV candidate and is currently studied in a phase 2 clinical trial in Mali (ClinicalTrials.gov ID NCT03917654). Pfs230D1-EPA contains an N-terminal fragment of the gamete surface protein Pfs230, including a part of the Pro-domain and the first of fourteen 6-cysteine (6-Cys, also known as cysteine motif) domains (Fig. 1a)^3,4^. Preclinical studies with a range of Pfs230 fragments showed that only vaccines containing the Pfs230 Pro-domain and/or Domain 1 (Pfs230Pro-D1) were able to induce functional antibodies with transmission-reducing activity (TRA)^5–9^. Importantly, the vast majority of Pfs230-specific antibodies are dependent on human complement that is also present in the mosquito blood meal^4,10–13^, while some antibodies retain strong TRA in the absence of complement^14,15^. The observation that recombinant fragments covering Domains 2 up to 14 (Pfs230D2-D14) did not induce functional antibodies^5,7,8^ led to the current hypothesis that these domains do not contain functional epitopes. We recently challenged this hypothesis by showing that the functional murine monoclonal antibody (mAb) 2A2.2a, elicited against full-length Pfs230 in *P. falciparum* gametocyte extract, does not target Pfs230Pro-D1^16^; thus far, the targeted domain remains unknown. In line with this observation, another recent study identified murine mAbs that have TRA but do not recognize Pfs230Pro-D1^13^.

Here, we aimed to identify functionally relevant Pfs230 epitopes outside Pfs230Pro-D1. We selected the high affinity murine mAb 18F25 IgG1 (18F25.1), elicited against female gametes^17,18^ and showed that it binds to Pfs230-D7. Since mAb 18F25.1 is a non-complement-fixing subtype (IgG1) antibody, whereas all of the described Pfs230 antibodies with functional activity are complement-dependent, we generated a complement-fixing subtype variant, 18F25 IgG2a (18F25.2a). This mAb 18F25.2a induced complement-mediated gamete lysis and strongly reduced *Plasmodium* transmission in standard membrane feeding assays (SMFA). Together, we show that Pfs230D7 is a target for a potent transmission-blocking mAb and provide new leads for the development of Pfs230-based vaccines.

## Results

### mAb 18F25 binds Pfs230 Domain 7

To identify mAbs that target Pfs230D2-D14 (Fig. 1a), we screened murine mAbs, elicited against whole parasites and parasite extract, for binding to full-length Pfs230 and absence of binding to Pro-D1. We observed that mAb 18F25.1^18^ binds to a conformational epitope on native Pfs230 in gametocyte extract with recognition being lost after reduction of disulfide bonds (Fig. 1b). While mAb 18F25.1 binds full-length Pfs230, it failed to recognize recombinant Pfs230CMB (containing Pro-D1), thus indicating that it targets an epitope on Pfs230D2-D14 (Fig. 1b). Next, we mapped the target domain of mAb 18F25.1 using a panel of recombinant Pfs230 fragments expressed in the wheat germ cell-free system (Supplementary Fig. 1)^5^. We first examined binding to the Pro-domain and fragments containing four consecutive 6-Cys domains and found that mAb 18F25.1 only reacted with D5-D8 and to a lesser extent D7-D10 (Fig. 1c). This suggests that the majority of interactions are with residues in D7 and/or D8. In a western blot with double-domain fragments, mAb 18F25.1 exclusively bound to D7-D8 (Supplementary Fig. 1). Using single-domain constructs D7 and D8, we found that mAb 18F25.1 binds to D7 and not D8 (Fig. 1d). Thus, mAb 18F25.1 targets an epitope that is (primarily) located on Pfs230D7.

### Subclass switch of mAb 18F25 IgG1 to IgG2a

**Fig. 2:**
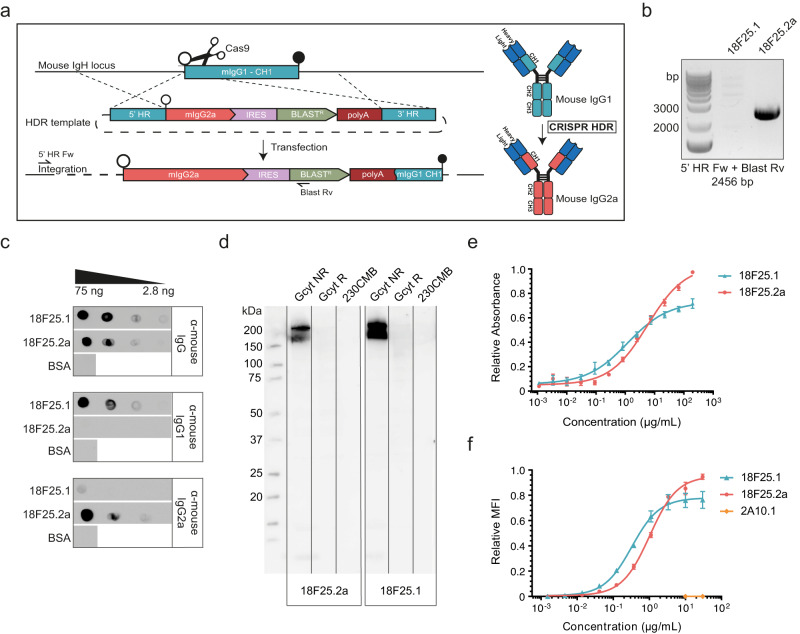
Generation of a subclass-switched 18F25 mAb using CRISPR/Cas9-based hybridoma engineering. **a** Schematic overview of the general strategy used to switch the isotype of 18F25 from IgG1 to IgG2a. A guide RNA directed Cas9 to make a double strand break in the CH1 domain of Ighg1 (gene locus), which was repaired by integration of the HDR template. This HDR template contained the coding sequence for the CH1-CH3 domains of Ighg2c (mIgG2a), Internal Ribosome Entry Site (IRES), Blasticidin resistance gene (BLAST^R^) and polyA signal sequence. The first 54 amino acids of CH1 from Ighg1 were removed after integration. HDR: Homology Directed Repair; HR: Homology Region. **b** Integration PCR on genomic DNA isolated from the 18F25.1 parental and the 18F25.2a-expressing hybridoma cell lines. The annealing sites of the primers are depicted in (**a**). **c** Dot blot with a titration (75, 25, 8.3 and 2.8 ng) of purified 18F25.1 and 18F25.2a antibody spotted on a nitrocellulose membrane. Three identical blots were subsequently incubated with α-mouse IgG, α-mouse IgG1 or α-mouse IgG2a secondary antibody. 75 ng bovine serum albumin (BSA) was included as negative control. **d** Western blots with non-reduced (NR) and reduced (R) *P. falciparum* NF54 gametocyte extract (Gcyt) and 20 ng of recombinant Pfs230CMB (230CMB). All samples were separated on one gel and transferred to a blot. The blot was cut in two parts that were incubated with mAb 18F25.1 or mAb 18F25.2a, as indicated. **e** mAb 18F25 recognition of native Pfs230 in *P. falciparum* NF54 gametocyte extract as assessed by ELISA. Values are means ± SEM from three independent experiments with three technical replicates each. Values from each experiment were normalized against an internal control to allow averaging across experiments. **f** mAb 18F25 binding to native Pfs230 on the surface of *P. falciparum* NF54 female gametes by flow cytometry analysis. Monoclonal antibody 2A10.1 (α-PfCSP) was included as negative control. Relative mean fluorescent intensities (MFI) are means ± SEM from two independent experiments with three technical replicates each. Values from each experiment were normalized against an internal control to allow averaging across experiments. The gating strategy is shown in Supplementary Fig. 3A.

mAb 18F25.1 binds to Pfs230D7, but fails to reduce transmission of *P. falciparum* to mosquitoes in SMFA in which cultured *P. falciparum* NF54 gametocytes were fed to *Anopheles stephensi* mosquitoes and TRA was determined using oocyst counts as readout^18^. The murine mAb 18F25.1 is a non-complement-fixing subtype IgG1 and we therefore switched the subtype to complement-fixing IgG2a by engineering the 18F25.1 hybridoma cell line using CRISPR/Cas9 (Fig. 2a). Using a guide RNA, Cas9 was directed to generate a double-stranded break in the constant heavy region 1 (CH1) of the *mIgG1* locus in the 18F25.1 hybridoma cell line. By simultaneously introducing a homology directed repair (HDR) template, the coding sequence of *mIgG1* CH1-CH3 was replaced by *mIgG2a* CH1-CH3 to generate a *mIgG2a* switch variant. Transfected cells were selected using blasticidin and subsequently a clonal cell line was established by limiting dilution. Correct genomic integration of the *mIgG2a* CH1-CH3 fragment in this cell line was verified by polymerase chain reaction (Fig. 2b) and subsequent Sanger sequencing. The cell line was expanded and expressed antibodies were purified from supernatant. Dot blots with subtype-specific detection antibodies confirmed that the modified cell line produced mIgG2a instead of mIgG1 (Fig. 2c). Next, we tested whether the specificity and affinity of mAb 18F25.2a was affected compared to the parental mAb 18F25.1. As expected, mAb 18F25.2a remained specific for Pfs230 in gametocyte extract (Fig. 2d). Furthermore, the affinity of 18F25.1 and 18F25.2a for Pfs230 in gametocyte extract were comparable (Fig. 2e). Binding of 18F25.1 and 18F25.2a to Pfs230 on the surface of live female gametes was similar with EC_50_ values of 0.32 µg/mL (95% CI [0.12, 0.52]) and 1.02 µg/mL (95% CI [0.7, 1.34]), respectively (Fig. 2f). Together these results demonstrate that we successfully generated a subclass switch of 18F25 from IgG1 to IgG2a while retaining its specificity and affinity.

### mAb 18F25.2a strongly reduces *P. falciparum* transmission to mosquitoes

**Fig. 3:**
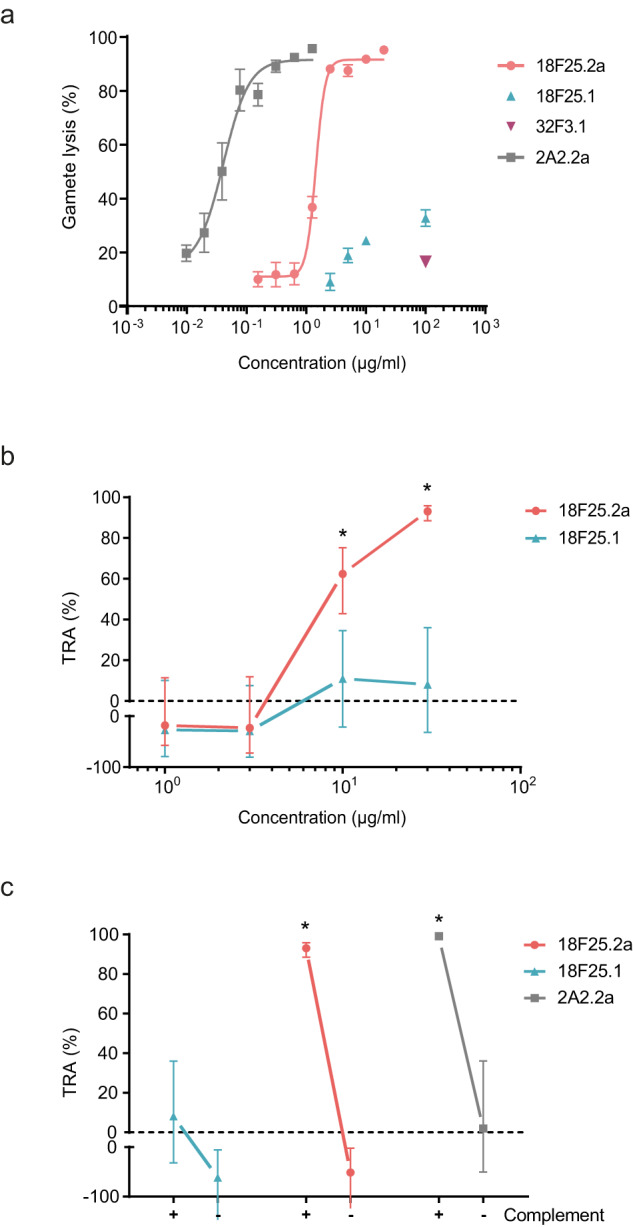
mAb 18F25.2a reduces *P. falciparum* transmission in a complement-dependent manner. **a** Flow cytometry assay to determine the capacity of mAbs 18F25.1 and 18F25.2a to lyse isolated female gametes in the presence of active human complement. The gating strategy is shown in Supplementary Fig. 3B. Complement-dependent mAb 2A2.2a (α-Pfs230) was included as positive control and complement-independent mAb 32F3.1 (α-Pfs48/45) as a negative control. Lysis percentages are presented as means ± SEM from two independent experiments with three technical replicates each. Values from each experiment were normalized against an internal control to allow averaging across experiments. 0% lysis is defined as the number of live gametes present after incubation with normal human serum only. **b** mAbs 18F25.1 and 18F25.2a were tested in two independent SMFAs with *P. falciparum* NF54 gametocytes and *A. stephensi* mosquitoes, in the presence of active human complement. TRA estimates are shown with 95% confidence intervals and were determined using a mixed-effects negative binomial regression model^43^. **c** mAbs 18F25.1 and 18F25.2a were tested at 30 µg/mL in SMFA, in the presence (+) and absence (−) of active human complement. Complement-dependent mAb 2A2.2a (α-Pfs230) was included as positive control. TRA (%) estimates are shown with 95% confidence intervals and were determined using a mixed-effects negative binomial regression model^43^. Asterisks indicate significant TRA (*p* < 0.05). Oocyst counts from individual SMFA experiments can be found in Supplementary Fig. 2.

Having generated a complement-fixing subclass variant of mAb 18F25, we next assessed its functional activity. We first tested the capacity of this mAb to lyse purified female gametes in vitro, in the presence of active human complement. 18F25.2a induced strong lysis of gametes; 10 µg/mL 18F25.2a was sufficient to lyse 91% of gametes, while the non-complement-fixing mAb 18F25.1 at the same concentration induced substantially less lysis (24%) (Fig. 3a). Next, we performed SMFAs to assess TRA in mosquitoes. Addition of mAb 18F25.1 to the infectious blood meal did not result in significant TRA (*p* > 0.23) across the concentrations tested (Fig. 3b), in agreement with previous findings^18^. In contrast, 10 and 30 µg/mL mAb 18F25.2a resulted in 62% (95% % CI [43, 75]) and 93% TRA (95% CI [89, 96]), respectively (Fig. 3b). These SMFA data strongly suggest that the capacity of the antibody to fix complement, determined by its subclass, is driving activity. To test whether the observed TRA is indeed complement-dependent, we also tested functional activity of mAbs in the presence of heat-inactivated complement. While 30 µg/mL 18F25.2a induced strong TRA in the presence of active complement, no significant TRA (*p* = 0.98) was observed when complement had been inactivated (Fig. 3c). Together these data show that mAb 18F25.2a is a functional mAb that potently reduces transmission of *P. falciparum* parasites to *A. stephensi* mosquitoes in a complement-dependent manner.

## Discussion

In this study we have uncovered a new functionally relevant Pfs230 domain. We show that murine mAb 18F25, elicited by immunization with whole female gametes, targets Pfs230D7. Furthermore, we demonstrate that the complement-fixing subtype mAb 18F25.2a blocks transmission to mosquitoes. After 30 years of research on this leading TBV candidate, these results identify Pfs230D7 as the first Pfs230 domain outside Pro-D1 that is targeted by a transmission-blocking mAb.

While TBVs hold promise as new tools for malaria elimination, very few candidates have been identified and tested (pre-)clinically^2^. Pfs25 was the first TBV candidate that was tested clinically, but despite promising results in rodents, it failed to induce potent and long-lasting TRA in humans^19^. Pfs230D1 and two Pfs48/45-based vaccines are currently being assessed in clinical studies (ClinicalTrials.gov ID NCT05400746)^20,21^, but it is not yet clear whether these can induce potent long-lasting TRA in humans. It is therefore important to continue discovery and development of novel TBV candidates. Here, we identify Pfs230D7 as a TBV candidate by showing that mAb 18F25 binds to a conformational epitope on Pfs230D7 and that it blocks transmission to mosquitoes in a complement-dependent manner.

Vaccines can ideally induce potent antibodies that target conserved epitopes. Although we characterized only one mAb against D7, thus providing limited insight in terms of potency, it is encouraging that mAb 18F25.2a is potent, showing 93% TRA at 30 µg/mL. We did not assess whether genetic variation in Pfs230D7 in circulating *P. falciparum* strains can affect the potency of mAb 18F25.2a. Interestingly, mAb 18F25 competed with murine mAb 11E3 which has TRA and recognizes *P. falciparum* isolates from geographically distinct areas^18^. This suggests that the functional target epitope of 18F25 may be conserved. So far, Pfs230 fragments that included D7 did not manage to induce TRA in mouse immunization studies^5,7–9^. One possible explanation could be that non-functional epitopes within these fragments are immunodominant, preventing the induction of sufficient functional antibodies for TRA, as previously observed for the 6-Cys domain protein Pfs47^22^. Another explanation could be that fragments containing D7 failed to induce functional antibodies since these were (largely) incorrectly folded. 6-Cys domain containing proteins, with up to three disulfide bonds per domain, are notoriously difficult to express in their native conformation. This is exemplified by the struggles to produce correctly folded Pfs48/45, another 6-Cys family member and leading TBV candidate, where correct folding is essential for induction of functional antibodies^23^. It is striking that all fragments containing D7 that were produced in the wheat germ cell-free system and were used in the current study to identify D7 as target of 18F25, failed to induce TRA after mice immunization^5^; these were recognized by conformational mAb 18F25 (Fig. 1), suggesting that some properly folded protein was present. We hypothesize that the majority of protein in these preparations may have been incorrectly folded and prevented the induction of a functional response. In future studies, mAb 18F25 may in fact be utilized to purify correctly folded protein, an approach that was instrumental in the preclinical development of a Pfs48/45-based vaccine^23^. When correctly folded Pfs230D7 is obtained, its potency should be assessed, and compared to Pfs230D1, in mouse immunization studies. In these studies, Pfs230D7 should also be combined with Pfs230D1 to explore whether including Pfs230D7 can enhance the efficacy of a Pfs230D1-based vaccine.

Almost all α-Pfs230 antibodies described to date are complement-dependent^4,10–13^. In agreement with this, our study shows that complement-fixing is the driving force behind the potency of 18F25.2a; this mAb does not reduce transmission in the absence of active complement, nor does the non-complement-fixing mAb 18F25.1 reduce transmission in the presence of complement (Fig. 3). The observed TRA is likely mediated by the lysis of gametes through formation of the membrane attack complex (MAC) as previously observed for Pfs230D1-specific antibodies^24,25^.

We used a CRISPR/Cas9-based method to modify a murine hybridoma cell line to produce complement-fixing subtype 18F25.2a instead of non-complement-fixing subtype 18F25.1. Similar methods have previously been used to engineer the genomic immunoglobulin loci of rat and mouse hybridoma cell lines^26–29^. In our study, we generated HDR and guide RNA plasmids that enable replacement of the constant region of *mIgG1* by that of *mIgG2a*, and as such these plasmids can be utilized to switch the subclass of any mouse hybridoma cell line from IgG1 to IgG2a. This method provides an attractive alternative approach to screening for spontaneous class switch mutants, a common method that has previously been used to generate the functional α-Pfs230 mAb 2A2.2a^30^.

mAbs induced against sexual stage parasites, containing native full-length proteins, are valuable tools for antigen discovery. Indeed, two mAbs allowed the identification of Pfs230 as a TBV candidate, although the binding site on Pfs230 of these mAbs is unknown^31^. We recently found that another Pfs230-specific mAb induced against parasites, 2A2.2a, only recognized and blocked transmission of a subset of *Plasmodium* isolates. Analysis of polymorphisms in the Pfs230 protein of the different isolates suggested that Pfs230D4 may be the target of this mAb, but we could not express recombinant Pfs230D4 to confirm D4-specificity^16^. Here, we demonstrate that mAb 18F25, also induced against parasites, binds to a conformational epitope within Pfs230D7 and blocks transmission. Many more murine mAbs have been generated against native Pfs230 in parasites^13,18,32^; some of these block transmission but do not compete with mAbs 2A2 or 18F25. It is therefore tempting to speculate that yet more Pfs230 domains are targets for functional antibodies and that mAbs induced against whole parasites can be used to identify these.

Overall, the identification of Pfs230D7 as a target for the potent mAb 18F25.2a has important implications for the future of Pfs230-based TBV research. Our study provides new incentive to investigate Pfs230D7 as well as other non-Pro-D1 domains of Pfs230 as potential TBV candidates.

## Methods

### Hybridoma cell line and general culture conditions

The parental mouse hybridoma line expressing the anti-Pfs230 mAb 18F25.1 has been generated from immunized BALB/c mice^17^ and characterized previously^18^. The cell line was maintained in Dulbecco’s Modified Eagle Medium (DMEM) (Gibco) supplemented with 1× MEM Non-Essential Amino Acids Solution (Gibco), 10 mM HEPES (Gibco), 10% heat-inactivated fetal bovine serum (FBS) (Gibco) and Penicillin-Streptomycin (Gibco). The cells were propagated by splitting (1:1) with fresh medium daily.

### Western blot

*P. falciparum* NF54 gametocyte extract was prepared as described previously^33^ and diluted to the equivalent of 500,000 gametocytes per well. For analysis under reducing conditions, a final concentration of 10 mM dithiothreitol (DTT) was added. Gametocyte extract and Pfs230CMB (aa 444–730)^34^ samples were mixed with 4× NuPAGE™ LDS sample buffer and heated for 10 min at 70 °C before loading on a 4–20% Bis-Tris gel (GenScript). 20 ng Pfs230CMB was loaded per well. Precision Plus Dual Color protein marker (Bio-Rad) was used as size standard. Proteins were transferred to a 0.45 µm nitrocellulose membrane using the Trans-Blot Turbo transfer system (Bio-Rad). The blots were blocked with 5% skimmed milk in PBS before incubation with 5 µg/mL 18F25.1, 5 µg/mL 18F25.2a or 1:5000 polyclonal serum from mice immunized with Pfs230Pro-D1 (aa 443–736)^35^. After washing, the strips were incubated with 1:3000 diluted polyclonal rabbit anti-Mouse IgG HRP (DAKO, Germany). Blots were developed with Clarity Max Western ECL substrate (Bio-Rad) and imaged on the ImageQuant™ LAS 4000 (GE Healthcare).

The gene sequences coding for the Pfs230 fragments were optimized for wheat codon usage, purchased from GenScript, and cloned into a pEU-E01-GST expression vector (CellFree Science, Matsuyama, Japan)^5^. The recombinant Pfs230 proteins were expressed as N-terminally GST-tagged proteins in wheat germ cell-free extract WEPRO7240G (CellFree Science) and purified by single-step GST-tag purification with a glutathione-Sepharose 4B column (Cytiva) using a robotic automated protein synthesizer Protemist DTII (CellFree Science) following manufacturer’s instructions. The purified proteins were mixed with SDS-sample buffer and β-mercaptoethanol^36^, and denatured at 37 °C for 30 min before loading on a 12.5% PAGE Tris gel (ATTO, Tokyo, Japan). 3.8 pmol of the proteins was loaded per well and Precision Plus Protein All blue standard (Bio-rad) was used as the size standard. Proteins were transferred to an Amersham Hybond P Low fluorescence 0.2 µm PVDF membrane (Cytiva) using the Trans-blot SD semidry transfer cell (Bio-rad). The blots were blocked with 5% skimmed milk in PBST before incubation with 1 µg/mL 18F25.1. After washing, the blots were incubated with 1:10,000 diluted polyclonal Sheep anti-Mouse IgG HRP (Cytiva). Blots were developed with Immobilon Western chemiluminescent HRP substrate (Millipore, MA) and imaged on the LAS-4000 (FUJIFILM, Tokyo, Japan). Unprocessed and uncropped scans of the western blots can be found in the Source data file.

### Plasmid design and cloning

The genome of BALB/c mice was extracted from the BALB_cJ_V1 Ensembl Browser (accession number: GCA_ 001632525.1). The *Ighg2c* sequence coding for the mIgG2a immunoglobulin heavy constant region (accession number: MGP_BALBcJ_G0000005) was used for the HDR plasmid. The gene sequence was codon-optimized for mouse expression, flanked with BmsBI-v2 restriction sites and synthetized by GeneArt (Invitrogen) (Supplementary Table 2). For the generation of the HDR plasmid, DNA was extracted from the mAb 18F25.1 producing hybridoma cell line using the Zymo Quick DNA Miniprep Plus Kit (D4068S). The 5’ and 3’ homology regions (HR) were amplified by polymerase chain reaction (PCR) using Platinum™ Taq High Fidelity (Invitrogen). The 5’ HR was designed to contain the native splice acceptor region of the first exon of the *Ighg1* gene (MGP_BALBcJ_G0000007), and thereafter the *Ighg2c* coding sequence. The internal ribosome entry site (IRES), blasticidin resistance gene (Bsr) and PolyA sequence were PCR amplified from a donor plasmid kindly provided by M. Verdoes^29^. During PCR, BmsBI-v2 restriction sites and suitable overhangs were added to all amplicons. An overview of the used primers is provided in Supplementary Table 3. The different DNA fragments were incorporated in the pGGAselect entry vector using the NEB® Golden Gate Assembly Kit (BsmBI-v2) following the manufacturer’s protocol (60 cycles).

To generate the guide RNA/Cas9, the pX330-U6-Chimeric_BB-CBh-hSpCas9 (gift from Feng Zhang; Addgene plasmid #42230)^37^ was used as an entry vector. Suitable guide RNA sequences in the first exon of *Ighg1* were obtained using the CCTop Software^38^. The 5’-GGTCACCATGGAGTTAGTTT-3’ guide was selected based on location, predicted off-targets and the CRISPRater efficacy score. The guide sequence was ordered as complementary single-strand DNA oligos with the appropriate BbsI- overhangs. After phosphorylation using the T4 PNK enzyme (New England Biolabs) by incubation at 37 °C for 30 min, the oligonucleotides were annealed by incubating the mixture at 95 °C for 5 min followed by gradually cooling to 25 °C with an increment of −0.2 °C/s. Annealed oligos were inserted in the BbsI-digested entry vector by ligation using T4 ligase (New England Biolabs).The guide RNA/Cas9 and HDR plasmids were isolated from Stellar™ *E. coli* cells (TaKaRa Bio, Japan) using the HiPure filter kit (Invitrogen) according to manufacturer’s protocol. The plasmids were verified by Sanger sequencing (Baseclear, Leiden).

### Hybridoma transfection

18F25.1 hybridoma cells, with a minimum viability of 90%, were used for nucleofection using the SF Cell Line 4D-Nucleofector X Kit L (V4XC-2020, Lonza). Briefly, cells were centrifuged, washed with PBS with 1% FBS and counted. One million cells were resuspended in the supplied SF medium containing 1 µg of HDR plasmid and 1 µg guide RNA/Cas9 plasmid, or 2 µg GFP plasmid (negative control). The cell suspensions were transferred to cuvettes and nucleofection was performed with the 4D-Nucleofection system (Lonza, CQ-104, Program SF). The cells were transferred to 6-wells plates with 3 mL of pre-warmed complete medium. Three days after transfection, the cells were transferred to 10 cm Petri dishes with 7 mL of complete medium supplemented with 7 µg/mL blasticidin (Invivogen, ant-bl-05). Cultures were kept on blasticidin until GFP-transfected hybridoma cells were dead, and HDR-transfected cells were confluent with a viability above 80%. Antibiotic-resistant cells were seeded at 0.3 cells per well in five round bottom 96-well plates in 100 µL complete medium (without blasticidin) for clonal expansion.

### Clone selection

After 10 days of culture, supernatant from wells with high cell densities (110 clones) were analyzed for IgG1 and IgG2a expression by dot blot (described below). IgG2a-expressing clones were selected for further characterization.

Genomic DNA from the parental hybridoma line and the selected hybridoma clones was isolated using the Zymo Quick DNA Miniprep Plus Kit (D4068S). To confirm integration, PCRs were performed using the polymerase kit PrimeSTAR GXL DNA (TaKaRa Bio, Japan) following manufacturer’s instructions. PCR was performed with a forward primer (5’ HR forward) annealing upstream of the 5’ end of the HDR template and a reverse primer annealing on the blasticidin gene (Blasticidin reverse) (Supplementary Table 3). Another PCR was performed using a forward primer annealing on the blasticidin gene (Blasticidin forward) and a reverse downstream of the 3’ homology region (3’HR reverse). There was overlap between the amplicons in order to cover the full integration site. The amplicons were visualized on a 1% agarose gel and the sequence was validated by Sanger sequencing (Baseclear, Leiden).

Finally, the clone that showed correct integration, high IgG2a expression levels and proper cell growth, was selected. The culture of this clone was expanded for antibody production.

### Antibody production

Hybridoma cells were left for 9 days without the addition of fresh medium for optimal mAb production. Supernatants from the parental hybridoma (18F25.1) and the engineered clone (18F25.2a) were centrifuged at 3000 × *g* for 10 min. Supernatants were loaded on 5 mL MabSelect™ Xtra columns (Cytiva) and antibodies were eluted using Glycine/HCL (pH2.5) buffer. The pH of peak fractions was immediately neutralized by adding Tris buffer (pH 8.8) to a final concentration of 0.1 M. The fractions were pooled, buffer exchanged to PBS with 1 mg/mL trehalose and subsequently freeze-dried (Martin Christ GmbH, Germany). Antibody concentration was determined at 280 nm on a NanoDrop™ 2000 spectrophotometer (ThermoScientific) assuming an extinction coefficient of 1.34.

Briefly, anti-Pfs230 mAb 2A2.2a has been derived from mice immunized with *P. falciparum* NF54 gametocyte extract and underwent spontaneous subclass switched as described previously^30,39^. Anti-CSP mAb 2A10.1 was produced by culturing the mice hybridoma cell line, as described above^40^.

### Dot blot

Dot blots were used to determine the mAb subclass. Two µL of each dilution of mAb was spotted on a 0.45 µm nitrocellulose membrane (Bio-Rad). The blots were dried for 30 minutes and subsequently blocked for one hour with 5% skimmed milk in PBS. The blots were washed three times with PBS with 0.05% Tween (PBST) before incubation with 1:2000 (in PBST) polyclonal rabbit Anti-Mouse IgGs HRP (Dako, P0260), Anti-Mouse IgG1 HRPO (Sigma, SAB3701171) or Anti-Mouse IgG2a HRPO (Sigma, SAB3701178) (in PBST). The blots were washed three times with PBST and once with PBS before incubation with Clarity™ Western ECL substrate (Bio-Rad). Imaging was performed on the ImageQuant™ LAS 4000 (GE Healthcare). The images of the blots were cropped and aligned. Unprocessed and uncropped scans of the dot blots can be found in the Source data file.

### Gametocyte ELISA

*P. falciparum* NF54 gametocyte extracts were prepared as described previously^33^. Nunc MaxiSorp™ 96-wells plates (ThermoFisher) were coated overnight at 4 °C with 100 µL lysate per well, equivalent to 75,000 gametocytes. Plates were blocked with 5% skimmed milk in PBS, incubated with a threefold dilution series of primary antibody starting at 50 µg/mL (in PBS) and detected with 1:3000 (in PBS) dilution polyclonal rabbit anti-Mouse IgG HRP (DAKO, P0260). The ELISA was developed by adding 100 µL tetramethylbenzidine (TMB). The color reaction was stopped by adding 50 µL 0.2 M H_2_SO_4_ and the optical density was read at 450 nm on an iMark™ microplate absorbance reader (Bio-Rad).

### Gamete purification

*N*-acetyl glucosamine treated 16-day old *P. falciparum* NF54 gametocyte cultures were centrifuged for 10 min at 2000 × *g* and resuspended in FBS using a volume that equals half the culture volume. Gametocytes were activated on a roller bank for 45 min at room temperature and thereafter centrifuged for 10 min at 2000 × *g* at 4 °C. The pellet was resuspended in 1 mL PBS, loaded onto a 7 mL layer of 11% w/v Accudenz (Accurate Chemical) and centrifuged for 30 min at 7000 × *g* at 4 °C without brake (Sorvall RC-5B Superspeed Centrifuge with HB-4 swing-out rotor). The top layer containing female gametes was collected, transferred to a 50 mL tube and filled up to 50 mL with PBS. Gametes were pelleted by centrifugation for 5 min at 2000 × *g* at 4 °C. They were resuspended in 1 mL PBS and counted using a Bürker-Turk counting chamber.

### Gamete binding and lysis assays

Incubations were carried out in PBS supplemented with 2% FBS and 0.02% sodium azide. 50,000 purified gametes per well in a V-bottom non-treated 96-well plate (Costar) were incubated for 1 h at room temperature with mAbs. In the case of a lysis assay, there is an addition of 20% normal human serum and incubation for 30 min at room temperature. Plates were centrifuged at 2000 × *g* for 3 min at 4 °C and washed three times with PBS. Gametes were then incubated with either 1:200 Alexa Fluor™ 488 Chicken anti-mouse IgG (H + L) (Invitrogen) (binding assay) or 1:200 anti-Pfs47 (rat mAb 47.1)^41^ labeled with DyLight™ 650 NHS ester (Thermo Scientific, Cat. No. 62266) (lysis assay). 1:1000 eBioscience™ Fixable Viability Dye eFluor™ 780 (Invitrogen, Cat. No. 65-0865-14) was added and gametes were incubated for another 30 min at room temperature. After three rounds of washing with PBS, samples were resuspended in PBS. mAb binding to gametes and gamete lysis were assessed by flow cytometry by analyzing a minimum of 2000 gametes with the Gallios™ 10-color system (Beckman Coulter) and analyzed with FlowJo (BD, version 10.7.1).

### SMFA

18F25.1 and 18F25.2a antibodies were diluted in FBS and mixed with mature *P. falciparum* NF54 gametocytes and human serum that contains active complement. In conditions with inactive complement, the human serum was heated for 30 min at 56 °C prior to mixing with the gametocytes. The blood meals were fed to *Anopheles stephensi* mosquitoes from a colony maintained at Radboudumc (Nijmegen, the Netherlands), as described previously^42^. Unfed and partially fed mosquitoes were removed. Mosquito midguts from 20 mosquitoes per condition were dissected 6–8 days after the blood meal. Midguts were stained with mercurochrome and oocysts were counted. TRA was defined as the reduction in oocyst intensity (oocysts per mosquito midgut) in a test condition compared to a negative control in which no antibody (FBS control) was added. We fitted a mixed-effects negative binomial regression model to the data and used this to estimate the TRA as previously described^43^. All samples were tested in two independent SMFA experiments for which the data are shown in Supplementary Fig. 2.

### Statistical analysis

Transmission reducing activity (TRA) was calculated as the reduction in oocysts compared to a negative control, using a negative binomial regression model as previously described^43^. SMFA data analyses were done in R (version 4.1.2).

### Reporting summary

Further information on research design is available in the Nature Research Reporting Summary linked to this article.

### Supplementary information

Supplementary Information

REPORTING SUMMARY

### Source data

Source Data file

## Data Availability

The authors declare that the data supporting the findings of this study are available within the paper and its Supplementary File.

## Materials availability

Monoclonal antibodies and plasmids generated in this study are available upon request.
