# Supplementary material for: Pfs230 Domain 7 is targeted by a potent malaria transmission-blocking monoclonal antibody
Source: NPJ Vaccines. 2023 Dec 12;8:186. doi: 10.1038/s41541-023-00784-x (PMC10716117; doi:10.1038/s41541-023-00784-x)
Supplement: Supplementary file 1 — Supplementary Information [file 41541_2023_784_MOESM1_ESM.pdf]

Pfs230 Domain 7 is targeted by a potent malaria transmission-blocking monoclonal antibody

Supplementary Information:
Supplementary Tables 1-3
Supplementary Figures 1-3

Maartje R. Inklaar[1], Roos M. de Jong[1], Ezra T. Bekkering[1], Hikaru Nagaoka[2], Felix L. Fennemann[3], Karina Teelen[1], Marga van de Vegte-Bolmer[1], Geert-Jan van Gemert[1], Rianne Stoter[1], C. Richter King[4], Nicholas I. Proellochs[1], Teun Bousema[1], Eizo Takashima[2], Takafumi Tsuboi[5], Matthijs M. Jore[1*]

[1] Department of Medical Microbiology, Radboudumc, Nijmegen, the Netherlands
[2] Division of Malaria Research, Proteo-Science Center, Ehime University, Matsuyama, Japan
[3] Department of Tumor Immunology, Radboudumc, Nijmegen, The Netherlands
[4] PATH's Center for Vaccine Innovation and Access, Washington, DC 20001, USA
[5] Division of Cell-Free Sciences, Proteo-Science Center, Ehime University, Matsuyama, Japan

Underlined authors contributed equally.
* corresponding author: Matthijs M. Jore (Matthijs.Jore@radboudumc.nl)

KEY WORDS: *Plasmodium falciparum*, Pfs230, monoclonal antibody, transmission-reducing activity

Supplementary Table 1. Overview of recombinant Pfs230 fragments used or mentioned in this study. All constructs were produced in the wheat-germ cell free system[1,2] with the exception of the plant-produced Pfs230CMB[3] and the *Lactococcus lactis*-produced Pfs230Pro-D1 (previously called Pfs230Pro+I)[4].

Name fragment	Amino acid boundaries
Pro	22-588
D1-D4	443-1274
D3-D6	910-1560
D5-D8	1280-2051
D7-D10	1690-2393
D9-D12	2052-2830
D11-D14	2448-3135
D1-D2	443-904
D3-D4	910-1274
D5-D6	1280-1560
D7-D8	1690-2051
D9-D10	2052-2393
D11-D12	2448-2830
D13-D14	2831-3135
D7	1690-1907
D8	1908-2051
Pfs230CMB	444-730
Pfs230Pro-D1	444-736
Pfs230C	443-1132

Supplementary Table 2. Overview of sequences used to generate the homology directed repair plasmid for CRISPR/Cas9-based engineering of the mouse hybridoma cell line. Red nucleotides correspond to the original 50 nucleotides preceding the *Ighg2c* gene in the BALB/c genome. Nucleotides that are underlined and bold indicate the first complete codon of *Ighg2c*. The two preceding cytosines form a codon with a guanine originating from the VDJ domains after RNA splicing.

mIgG2a synthetic gene	cagtcgtctcagctagcATCTGAGGCCACAGATAACAGAAAAGCTCACACATCCTCCTCTCTTGCAGCC**AAG**ACCACCGCTCCTAGCGTGTACCCT<br>CTGGCTCCTGTGTGTGGCGACACAACAGGCAGCTCTGTGACACTGGGCTGTCTGGTCAAGGGCTACTTCCCCGAACCAGTGACACTGACCTGGAAC<br>AGCGGCTCTCTGTCTAGCGGCGTGCACACATTTCCAGCCGTGCTGCAGAGCGACCTGTACACACTGTCCAGCAGCGTGACCGTGACCAGCAGCACA<br>TGGCCTAGCCAGAGCATCACCTGTAACGTGGCCCATCCTGCCAGCTCCACCAAGGTGGACAAGAAGATCGAGCCTAGAGGCCCCACCATCAAGCCC<br>TGTCCTCCATGCAAATGCCCCGCTCCTAATCTGCTCGGCGGACCCAGCGTGTTCATCTTCCCACCTAAGATCAAGGACGTGCTGATGATCTCTCTG<br>AGCCCCATCGTGACCTGCGTGGTGGTGGATGTGTCTGAGGACGACCCTGACGTGCAGATCAGTTGGTTCGTGAACAACGTGGAAGTGCACACAGCC<br>CAGACACAGACCCACAGAGAGGACTACAACAGCACCCTGAGAGTGGTGTCTGCCCTGCCTATCCAGCACCAGGATTGGATGAGCGGCAAAGAATTC<br>AAGTGCAAAGTGAACAACAAGGACCTGCCTGCTCCTATCGAGAGAACCATCAGCAAGCCCAAGGGCTCTGTCAGGGCTCCTCAGGTGTACGTTCTG<br>CCACCTCCTGAGGAAGAGATGACCAAGAAACAAGTGACCCTCACCTGTATGGTCACCGACTTCATGCCCGAGGACATCTACGTGGAATGGACCAAC<br>AACGGCAAGACCGAGCTGAACTACAAGAACACCGAGCCTGTGCTGGACTCCGACGGCAGCTACTTCATGTACAGCAAGCTGCGCGTCGAGAAGAAG<br>AACTGGGTCGAGAGAAACAGCTACAGCTGCAGCGTGGTGCACGAGGGACTGCACAACCACCACACCACCAAGAGCTTCAGCAGAACCCCTGGCAAA<br>TGAgccggcatgagacggcat
5’HR	GTTTGTGTATAGGCAAGAAGTGAATCCTGACCCAAGAATAGAGAGTGCTAAACGGACTTAGCTCAAAGACAACTGAAAAAGACAATGCCTGCAAAA<br>CAAAGCTAAGGCCAGAGCTCTTGGACTATGAAGAGTTCAGGGAACCTAAGAACAGGGACCATCTGTGTACAGGCCAAGGCCGGTAGAAGCAGCCTA<br>GGAAATGTCAAGAGCCAACGTGGCTGGGTGGGCAAAGACAGGAAGGGACTGTTAGGCTGCAGGGATGTGCCGACTTCAATTTGTGCTTCAGTGTTG<br>TCCAGATTGTGTGCAGCCATATGGCCCAGGTATAAGAAGTTTAACAGTGGAACACAGATGCCCACATCAGACAGCTGGGGGGTGGGGGGGGTGAAC<br>ACAGATACCCATACTGGAAAGCAGGTGGGGCATTTTCCTAGGAACGGGACTGGGCTCAATGGCCTCAGGTCTCATCTGGTCTGGTGATCCTGACAT<br>TGACAGGCCCAAATGTTGGATATCACCTACTCCATGTAGAGAGTCGGGGACATGGGAAGGGTGCAAAAGAGCGGCCTTCTAGAAGGTTTGGTCCTG<br>TCCTGTCCTGTCTGACAGTGTAATCACATATACTTTTTCTTGTAGCC
mIgG2a	AAGACCACCGCTCCTAGCGTGTACCCTCTGGCTCCTGTGTGTGGCGACACAACAGGCAGCTCTGTGACACTGGGCTGTCTGGTCAAGGGCTACTTC<br>CCCGAACCAGTGACACTGACCTGGAACAGCGGCTCTCTGTCTAGCGGCGTGCACACATTTCCAGCCGTGCTGCAGAGCGACCTGTACACACTGTCC<br>AGCAGCGTGACCGTGACCAGCAGCACATGGCCTAGCCAGAGCATCACCTGTAACGTGGCCCATCCTGCCAGCTCCACCAAGGTGGACAAGAAGATC<br>GAGCCTAGAGGCCCCACCATCAAGCCCTGTCCTCCATGCAAATGCCCCGCTCCTAATCTGCTCGGCGGACCCAGCGTGTTCATCTTCCCACCTAAG<br>ATCAAGGACGTGCTGATGATCTCTCTGAGCCCCATCGTGACCTGCGTGGTGGTGGATGTGTCTGAGGACGACCCTGACGTGCAGATCAGTTGGTTC<br>GTGAACAACGTGGAAGTGCACACAGCCCAGACACAGACCCACAGAGAGGACTACAACAGCACCCTGAGAGTGGTGTCTGCCCTGCCTATCCAGCAC<br>CAGGATTGGATGAGCGGCAAAGAATTCAAGTGCAAAGTGAACAACAAGGACCTGCCTGCTCCTATCGAGAGAACCATCAGCAAGCCCAAGGGCTCT<br>GTCAGGGCTCCTCAGGTGTACGTTCTGCCACCTCCTGAGGAAGAGATGACCAAGAAACAAGTGACCCTCACCTGTATGGTCACCGACTTCATGCCC<br>GAGGACATCTACGTGGAATGGACCAACAACGGCAAGACCGAGCTGAACTACAAGAACACCGAGCCTGTGCTGGACTCCGACGGCAGCTACTTCATG<br>TACAGCAAGCTGCGCGTCGAGAAGAAGAACTGGGTCGAGAGAAACAGCTACAGCTGCAGCGTGGTGCACGAGGGACTGCACAACCACCACACCACC<br>AAGAGCTTCAGCAGAACCCCTGGCAAATGA
IRES Bsr polyA	GTCGAGGCCCCTCTCCCTCCCCCCCCCCTAACGTTACTGGCCGAAGCCGCTTGGAATAAGGCCGGTGTGCGTTTGTCTATATGTTATTTTCCACCA<br>TATTGCCGTCTTTTGGCAATGTGAGGGCCCGGAAACCTGGCCCTGTCTTCTTGACGAGCATTCCTAGGGGTCTTTCCCCTCTCGCCAAAGGAATGC<br>AAGGTCTGTTGAATGTCGTGAAGGAAGCAGTTCCTCTGGAAGCTTCTTGAAGACAAACAACGTCTGTAGCGACCCTTTGCAGGCAGCGGAACCCCC<br>CACCTGGCGACAGGTGCCTCTGCGGCCAAAAGCCACGTGTATAAGATACACCTGCAAAGGCGGCACAACCCCAGTGCCACGTTGTGAGTTGGATAG<br>TTGTGGAAAGAGTCAAATGGCTCTCCTCAAGCGTATTCAACAAGGGGCTGAAGGATGCCCAGAAGGTACCCCATTGTATGGGATCTGATCTGGGGC<br>CTCGGTGCACATGCTTTACATGTGTTTAGTCGAGGTTAAAAAACGTCTAGGCCCCCCGAACCACGGGGACGTGGTTTTCCTTTGAAAAACACGAT<br>GATAATATGGCCACAGAATTCGCCACCATGGCCAAGCCTTTGTCTCAAGAAGAATCCACCCTCATTGAAAGAGCAACGGCTACAATCAACAGCATC<br>CCCATCTCTGAAGACTACAGCGTCGCCAGCGCAGCTCTCTCTAGCGACGGCCGCATCTTCACTGGTGTCAATGTATATCATTTTACTGGGGGACCT<br>TGTGCAGAACTCGTGGTGCTGGGCACTGCTGCTGCTGCGGCAGCTGGCAACCTGACTTGTATCGTCGCGATCGGAAATGAGAACAGGGGCATCTTG<br>AGCCCCTGCGGACGGTGCCGACAGGTGCTTCTCGATCTGCATCCTGGGATCAAAGCCATAGTGAAGGACAGTGATGGACAGCCGACGGCAGTTGGG<br>ATTCGTGAATTGCTGCCCTCTGGTTATGTGTGGGAGGGCTAAGTACTAGTCGAGTGTGCCTTCTAGTTGCCAGCCATCTGTTGTTTGCCCCTCCCC<br>CGTGCCTTCCTTGACCCTGGAAGGTGCCACTCCCACTGTCCTTTCCTAATAAAATGAGGAAATTGCATCGCATTGTCTGAGTAGGTGTCATTCTAT<br>TCTGGGGGGTGGGGTGGGGCAGGACAGCAAGGGGGAGGATTGGGAAGACAATAGCAGGCATGCTGGGGATGCGGTGGGCTCTATGGAGATCTTGTA<br>CA
3’HR	CTAACTCCATGGTGACCCTGGGATGCCTGGTCAAGGGCTATTTCCCTGAGCCAGTGACAGTGACCTGGAACTCTGGATCCCTGTCCAGCGGTGTGC<br>ACACCTTCCCAGCTGTCCTGCAGTCTGACCTCTACACTCTGAGCAGCTCAGTGACTGTCCCCTCCAGCACCTGGCCCAGCGAGACCGTCACCTGCA<br>ACGTTGCCCACCCGGCCAGCAGCACCAAGGTGGACAAGAAAATTGGTGAGAGGACATATAGGGAGGAGGGGTTCACTAGAAGTGAGGCTCAAGCCA<br>TTAGCCTGCCTAAACCAACCAGGCTGGACAGCCATCACCAGGAAATGGATCTCAGCCCAGAAGATCAAAAGTTGTTCTTCTCCCTTCTGGAGATTT<br>CTATGTCCTTTACACTCATTGGTTAATATCCTGGGTTGGATTCCCACACATCTTGACAAACAGAGACAAATTTGAGTATCACCAGCCAAAAGTCAT<br>ACCCAAAAACAGCCTGGCATGACCTCACACCAGACTCAAACTTACCCTACCTTTATCCTGGTGGCTTCTCATCTCCAGACCCCAGTAACACATAGC<br>TTTCTCTCCACAGTGCCCAGGGATTGTGGTTGTAAGCCTTGCATATGTACAGGTAAGTCAGTAGGCCTTTCACCCTGACCCCAGATGCAACAAGTG<br>GCCATGTTGGAGGGTGGCCCAGGTATTGACCTATTTCCACCTTTCTTCTTCATCCTTAGTCCCAGAAGTATCATCTGTCTTCATCTTCCCCCCAAA<br>GCCCAAGGATGTGCTCACCATTACTCTGACTCCTAAGGTCACGTGTGTTGTGGTAGACATCAGC

Supplementary Table 3. Overview of primers used in this study.

**Plasmid Design**	
5’HR Forward	cagtcgtctcaggagtcgacGTTTGTGTATAGGCAAGAAGTGAATCCTGAC
5’HR Reverse	ctagcgtctcaTCTTGGCTACAAGAAAAAGTATATGTGATTACACTG
3’HR Forward	cagtcgtctctacccgggCTAACTCCATGGTGACCCTGGGATG
3’HR Reverse	ctagcgtctctatggaccggtGCTGATGTCTACCACAACACACGTGAC
IRES Bsr Poly A Forward	cagtcgtctcaggcaGTCGAGGCCCCTCTCCCTCC
IRES Bsr Poly A Reverse	ctagcgtctcagggtTGTACAAGATCTCCATAGAGCCCACC
mIgG2a Forward	cagtcgtctctAAGACCACCGCTCCTAGCGTG
mIgG2a Reverse	ATGCcgtctcATGCCGGCTC
**Integration PCR**	
5’HR Forward	GAGAACCAAGCTAAAAAGTTATGTCAAACCAC
Blasticidin Reverse	ATACATTGACACCAGTGAAGATGC
Blasticidin Forward	CAGCAGAACCCCTGGCAAATG
3’HR Reverse	CATCTACAAACCAGCTGAACTGGACC

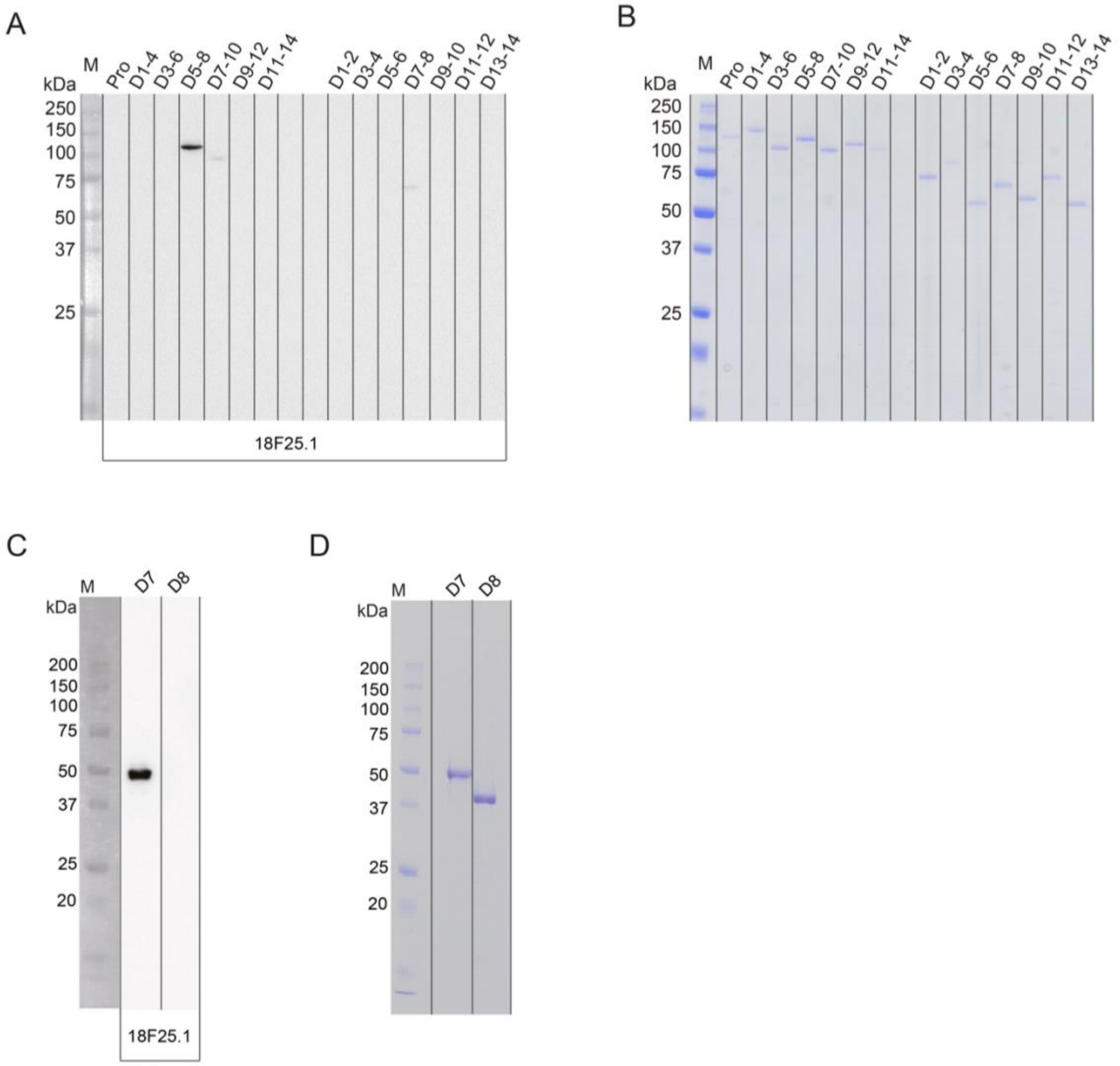

Supplementary Figure 1. 18F25 detection of Pfs230 recombinant fragments. **(A)** Western blot with mAb 18F25.1 and recombinant multi-domain Pfs230 fragments. The recombinant fragments were expressed with a glutathione S-transferase (GST) tag. **(B)** SDS-PAGE gel of the multi-domain wheat-germ cell free system produced recombinant multi-domain Pfs230 fragments used in (A). **(C)** Western blot with mAb 18F25.1 and recombinant single-domain Pfs230 fragments. (D) SDS-PAGE gel of the single domain wheat-germ cell free system produced recombinant Pfs230 fragments shown in (C). Note that part of the western blot in (A) and the western blot in (C) are also shown Figure 1, though for clarity are depicted here next to the SDS-PAGE results.

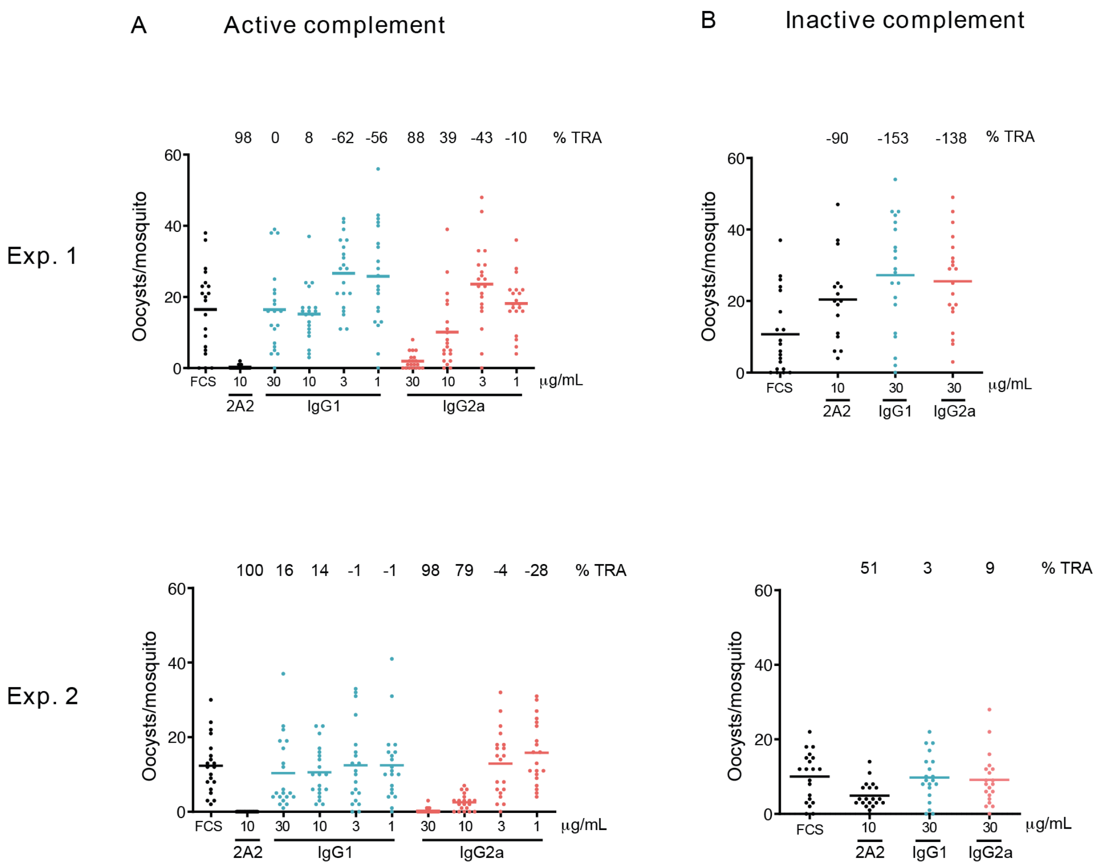

Supplementary Figure 2. Overview of individual standard membrane feeding assays (SMFA) that form the basis for reported TRA values in Figure 3. 18F25.1 (IgG1) and 18F25.2a (IgG2a) titrations were tested in two independent SMFAs with *P. falciparum* NF54 gametocytes and *A. stephensi* mosquitoes with active complement (A). The highest mAb concentration (30 µg/mL) was also tested in these assays with heat-inactivated complement (B). Complement-dependent α-Pfs230 monoclonal antibody 2A2.2a (2A2) was included as positive control in both SMFAs. Per condition, 20 individual mosquitoes were dissected and oocysts were counted. The line represents the mean number of oocysts per mosquito. The transmission reducing activity (% TRA) was calculated as the percentage in reduction in the number of oocysts per mosquito compared to the control in which no antibody was added (FCS control). Note that for 10 µg/mL 2A2.2a with inactivated complement in experiment 1, oocyst counts could be obtained for 16 mosquitoes instead of 20.

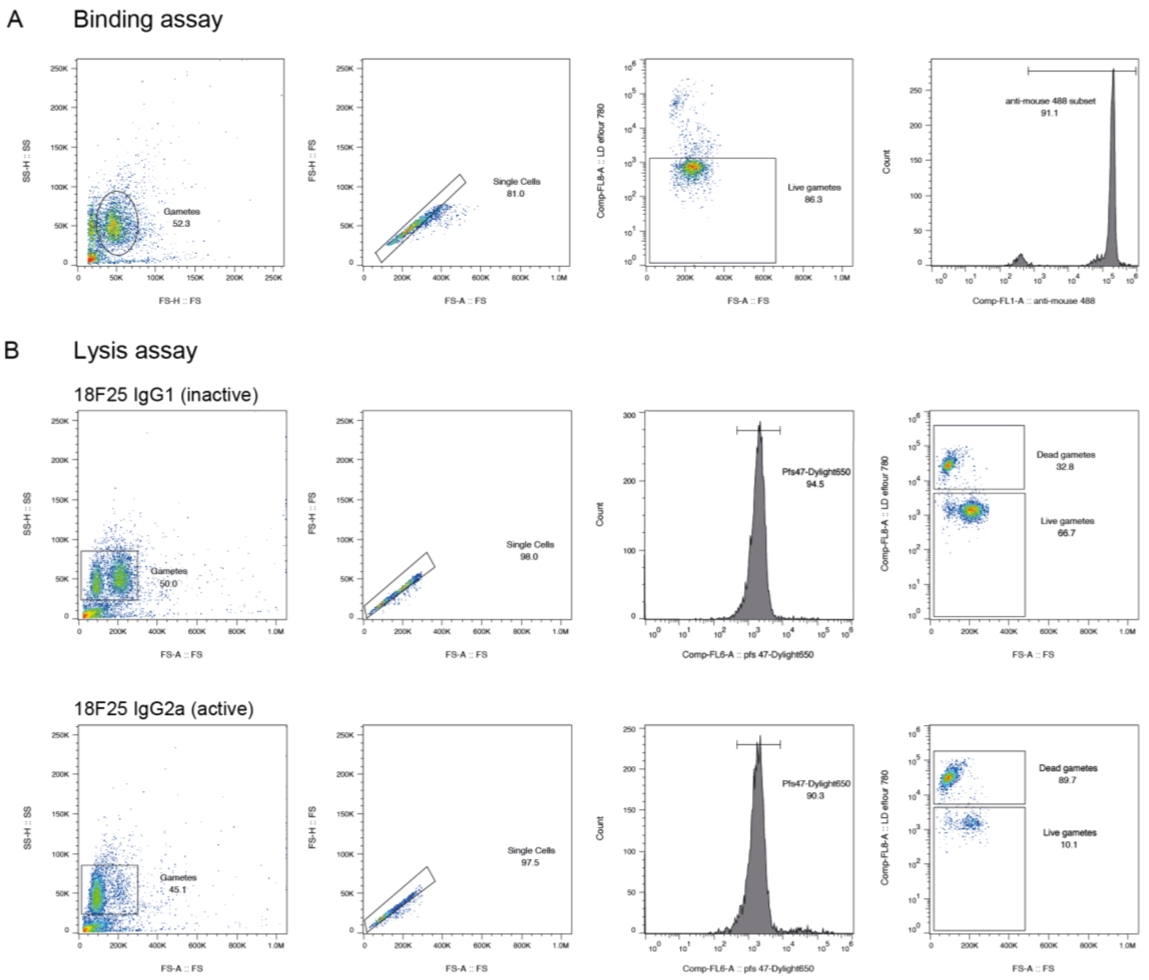

Supplementary Figure 3. Overview of flow cytometry gating strategies. Exemplary plots that provide an overview of the gating strategy for (A) a binding assay with live female gametes and (B) a lysis assay with live female gametes. In the binding assay (A) gametes were gated for single cells (2nd column) and then live gametes were selected based on the absence of live-dead stain LD efluor 780, which stains dead cells (3rd column). (B) lysis assay with an inactive mAb (IgG1, top row) and active mAb (IgG2a, bottom row). Gametes were gated for single cells (2nd column), and then for Pfs47 positivity. Dead gametes were stained with live-dead stain LD efluor 780 to determine the percentage dead cells (4th column). Note that two gamete populations can be observed in the forward scatter plots (1st column) in A and B; the left population contains dead gametes, the right population contains live gametes. In the binding assay (A) we gated only the live population, while in the lysis assay (B) we gated both live and dead populations. SS-H = side scatter height, SS = side scatter, FS-H = forward scatter height, FS-A = forward scatter area, LD = live dead.

Supplementary References

1 Tachibana, M. *et al.* Identification of domains within Pfs230 that elicit transmission blocking antibody responses. *Vaccine* **37**, 1799-1806, doi:10.1016/j.vaccine.2019.02.021 (2019).

2 Miura, K. *et al.* Functional comparison of Plasmodium falciparum transmission-blocking vaccine candidates by the standard membrane-feeding assay. *Infect Immun* **81**, 4377-4382, doi:10.1128/IAI.01056-13 (2013).

3 Farrance, C. E. *et al.* A plant-produced Pfs230 vaccine candidate blocks transmission of Plasmodium falciparum. *Clin Vaccine Immunol* **18**, 1351-1357, doi:10.1128/CVI.05105-11 (2011).

4 Singh, S. K. *et al.* Pfs230 and Pfs48/45 Fusion Proteins Elicit Strong Transmission-Blocking Antibody Responses Against Plasmodium falciparum. *Front Immunol* **10**, 1256, doi:10.3389/fimmu.2019.01256 (2019).
