# Supplementary material for: Pfs230 Domain 7 is targeted by a potent malaria transmission-blocking monoclonal antibody
Source: NPJ Vaccines. 2023 Dec 12;8:186. doi: 10.1038/s41541-023-00784-x (PMC10716117; doi:10.1038/s41541-023-00784-x)
Supplement: Supplementary file 2 — REPORTING SUMMARY [file 41541_2023_784_MOESM2_ESM.pdf]

nature portfolio

Corresponding author(s): Matthijs M. Jore

Last updated by author(s): Jun 6, 2023

Reporting Summary

Nature Portfolio wishes to improve the reproducibility of the work that we publish. This form provides structure for consistency and transparency in reporting. For further information on Nature Portfolio policies, see our Editorial Policies and the Editorial Policy Checklist.

Statistics

For all statistical analyses, confirm that the following items are present in the figure legend, table legend, main text, or Methods section.

n/a	Confirmed	
☐	☒	The exact sample size (*n*) for each experimental group/condition, given as a discrete number and unit of measurement
☐	☒	A statement on whether measurements were taken from distinct samples or whether the same sample was measured repeatedly
☐	☒	The statistical test(s) used AND whether they are one- or two-sided<br>*Only common tests should be described solely by name; describe more complex techniques in the Methods section.*
☒	☐	A description of all covariates tested
☒	☐	A description of any assumptions or corrections, such as tests of normality and adjustment for multiple comparisons
☐	☒	A full description of the statistical parameters including central tendency (e.g. means) or other basic estimates (e.g. regression coefficient) AND variation (e.g. standard deviation) or associated estimates of uncertainty (e.g. confidence intervals)
☒	☐	For null hypothesis testing, the test statistic (e.g. *F*, *t*, *r*) with confidence intervals, effect sizes, degrees of freedom and *P* value noted<br>*Give P values as exact values whenever suitable.*
☒	☐	For Bayesian analysis, information on the choice of priors and Markov chain Monte Carlo settings
☒	☐	For hierarchical and complex designs, identification of the appropriate level for tests and full reporting of outcomes
☒	☐	Estimates of effect sizes (e.g. Cohen's *d*, Pearson's *r*), indicating how they were calculated

Our web collection on statistics for biologists contains articles on many of the points above.

Software and code

Policy information about availability of computer code

Data collection	For flow cytometry assays, Kaluza v2.2.1 was used.
Data analysis	Graphpad Prism v9 was used for data analysis. FlowJo v10.7.1 was used for analyses of flow data

For manuscripts utilizing custom algorithms or software that are central to the research but not yet described in published literature, software must be made available to editors and reviewers. We strongly encourage code deposition in a community repository (e.g. GitHub). See the Nature Portfolio guidelines for submitting code & software for further information.

Data

Policy information about availability of data

All manuscripts must include a data availability statement. This statement should provide the following information, where applicable:

- Accession codes, unique identifiers, or web links for publicly available datasets
- A description of any restrictions on data availability
- For clinical datasets or third party data, please ensure that the statement adheres to our policy

The authors declare that all data supporting the findings of this study are provided in the main document and supplementary files

Research involving human participants, their data, or biological material

Policy information about studies with human participants or human data. See also policy information about sex, gender (identity/presentation), and sexual orientation and race, ethnicity and racism.

Reporting on sex and gender	N/A
Reporting on race, ethnicity, or other socially relevant groupings	N/A
Population characteristics	N/A
Recruitment	N/A
Ethics oversight	N/A

Note that full information on the approval of the study protocol must also be provided in the manuscript.

Field-specific reporting

Please select the one below that is the best fit for your research. If you are not sure, read the appropriate sections before making your selection.

☒ Life sciences ☐ Behavioural & social sciences ☐ Ecological, evolutionary & environmental sciences

For a reference copy of the document with all sections, see nature.com/documents/nr-reporting-summary-flat.pdf

Life sciences study design

All studies must disclose on these points even when the disclosure is negative.

Sample size	No sample sizes have been calculated as this was not relevant for the current study.
Data exclusions	No data has been excluded in this study.
Replication	Presented data of gamete binding and lysis assays are based on at least 2 independent assays with at least 2 technical replicates each. Antibody samples were tested in 2 independent SMFA experiments with 20 mosquitoes (observations) for each condition. Replicate numbers are stated in the figure legends.
Randomization	No randomization was applied in this study as this was not relevant.
Blinding	No animals or humans were involved in the study, therefore there was no need to use blinding in our experiment. Oocyst counting in SMFA is not subjective.

Reporting for specific materials, systems and methods

We require information from authors about some types of materials, experimental systems and methods used in many studies. Here, indicate whether each material, system or method listed is relevant to your study. If you are not sure if a list item applies to your research, read the appropriate section before selecting a response.

Materials & experimental systems

n/a	Involved in the study	
☐	☒	Antibodies
☐	☒	Eukaryotic cell lines
☒	☐	Palaeontology and archaeology
☒	☐	Animals and other organisms
☒	☐	Clinical data
☒	☐	Dual use research of concern
☒	☐	Plants

Methods

n/a	Involved in the study	
☒	☐	ChIP-seq
☐	☒	Flow cytometry
☒	☐	MRI-based neuroimaging

Antibodies

Antibodies used	The newly generated 18F25.2a is thoroughly described in the paper. Non-commerical mAbs (2A2 and 2A10) are described in the methods section and include references.

	Commercial antibodies include polyclonal Sheep anti-Mouse IgG HRP (Cytiva), polyclonal rabbit anti-Mouse IgG HRP (DAKO, Germany), polyclonal rabbit Anti-Mouse IgGs HRP (Dako, P0260), Anti-Mouse IgG1 HRPO (Sigma, SAB3701171), Anti-Mouse IgG2a HRPO (Sigma, SAB3701178), Alexa Fluor™ 488 Chicken anti-mouse IgG (H+L) (Invitrogen), anti-Pfs47 (rat mAb 47.1) 34 labelled with DyLight™ 650 NHS ester (Thermo Scientific, Cat. No. 62266),
Validation	Antigen-specificity for non-commercial antibodies was confirmed by western blot. Commercial antibodies were tested by dot blot and/or solid-phase adsorbed to ensure minimal cross-reactivity.

Eukaryotic cell lines

Policy information about cell lines and Sex and Gender in Research

Cell line source(s)	Plasmodium falciparum NF54 has been adapted to culture and cloned at Radboudumc and previously been published. A hybridoma of 18F25.1 has been generated at Radboudumc and was published previously. Generation of 18F25.2a is described in the current manuscript.
Authentication	Clonality and identity of Plasmodium falciparum NF54 has been confirmed by PCR on hypervariable genes (GLURP, MSP1, MSP2). Hybridoma authentication has been conducted by characterisation of generated monoclonal antibodies (western/ dot blots and ELISA to determine specificity)
Mycoplasma contamination	Hybridoma cell lines have been tested for mycoplasma contamination, with negative results.
Commonly misidentified lines (See ICLAC register)	N/A

Flow Cytometry

Plots

Confirm that:

☐ The axis labels state the marker and fluorochrome used (e.g. CD4-FITC).

☒ The axis scales are clearly visible. Include numbers along axes only for bottom left plot of group (a 'group' is an analysis of identical markers).

☐ All plots are contour plots with outliers or pseudocolor plots.

☒ A numerical value for number of cells or percentage (with statistics) is provided.

Methodology

Sample preparation	Female gametes (Plasmodium falciparum) were isolated using a Nycodenz layer and stained as described in detail in the Methods section.
Instrument	Gallios™ 10-color system (Beckman Coulter)
Software	FlowJo v10.7.1 (BD)
Cell population abundance	No cell sorting was performed.
Gating strategy	Starting with FSH::FS/SSH::FS to select the gamete population, then FSA::FS/FSH::FS to select the single cells in the diagonal line of the plot. Subsequently, an FSA::FS/LD780 plot with a cutoff for live cells at 10^3. In the case of a lysis assay (see Methods), a histogram with anti-Pfs47-650 (FL-6) determines the definite gamete population. For a binding assay (see Methods) a histogram of anti-mouse 488 (FL-1) was used to determine mean fluorescent intensity.

☒ Tick this box to confirm that a figure exemplifying the gating strategy is provided in the Supplementary Information.
